# Characterization of a novel HIV-1 unique recombinant form between CRF07_BC and CRF55_01B in men who have sex with men in Guangzhou, China

**DOI:** 10.1371/journal.pone.0175770

**Published:** 2017-04-12

**Authors:** Yue Wu, Xuqi Ren, Dan Yin, Haiying Wang, Zhengwei Wan, Xiufen Li, Guifang Hu, Shixing Tang

**Affiliations:** 1 Guangdong Provincial Key Laboratory of Tropical Disease Research, Department of Epidemiology, School of Public Health, Southern Medical University, Guangzhou, Guangdong, China; 2 Dermatology Hospital of Southern Medical University, Guangzhou, China; 3 Guangdong Provincial Dermatology Hospital, Guangzhou, China; China Academy of Chinese Medical Sciences, CHINA

## Abstract

Here, we report the genetic diversity of HIV-1 and emergence of novel HIV-1 unique recombinant forms (URF) in both HIV-infected intravenous drug users (IDU) and men who have sex with men (MSM) in Guangzhou, China. We further characterized a novel URF strain isolated from an HIV-infected MSM, GD698. Near full-length genome (NFLG) phylogenic analysis showed that this novel URF was composed of CRF07_BC and CRF55_01B, with two recombinant breakpoints (nt 6,003 and 8,251 relative to the HXB2 genome) in the *vpu/env* and *env* genes, respectively. Twenty six percent of the genome is classified as CRF55_01B, spanning part of *vpu* and most of the *env* gene. The remaining 74% of the genome is classified as CRF07_BC. Both the backbone CRF07_BC sequence and CRF55_01B fragment were clustered with the HIV-1 isolates found in MSM. The emergence of the novel HIV-1 recombinant indicates the ongoing recombinants derived from the CRF07_BC and CRF55_01B isolates, and provides critical insights into our understanding of the dynamics and complexity of the HIV-1 epidemic in China.

## Introduction

One of the characteristics of human immunodeficiency virus type 1 (HIV-1) is its extremely high level of genetic variation, which results in the existence of four groups: M, O, N, and P. Within HIV-1 group M, there are nine subtypes (A, B, C, D, F, G, H, J, and K) and six derivatives (A1-A4 and F1, F2). Moreover, to date, co-infection and recombination of different HIV-1 genotypes have resulted in emergence of as many as 88 circulating recombinant forms (CRFs) (https://www.hiv.lanl.gov/content/sequence/HIV/CRFs/CRFs.html) and numerous unique recombinant forms (URFs). Furthermore, third generation HIV-1 recombinant forms have been identified as the result of recombination between various HIV-1 CRFs, such as the CRF30_0206 variant.

In the past 20 years, the predominant HIV-1 genotypes have been changing in China. Subtype B’ (the Thailand variant of subtype B)/B, CRF01_AE, CRF07_BC, and CRF08_BC are becoming dominant [1, 2]. Several additional recombinant forms were reported in China, including CRF07_BC [3], CRF08_BC [4], CRF55_01B [5], CRF57_BC [6], CRF59_01B [7], CRF61_BC, CRF62_BC [8], CRF64_BC [9], and CRF65_cpx [10], CRF67_01B, CRF68_01B [11] and CRF78_cpx [12]. Furthermore, sexual contact has become a major transmission route in China, in particular by the prevalence of anal intercourse in the population of men who have sex with men (MSM) [13]. Continuous emergence of HIV-1 URFs in MSMs is a major challenge for preventing the spread of the HIV-1 epidemic. To date, several URFs consisting of CRF01_AE, CRF07_BC and CRF55_01B have been reported in MSMs in China [14–16].

To monitor the genetic diversity of HIV-1 and the emergence of new recombinants, HIV-1 genotypes and possible novel HIV-1 URFs were determined in HIV-infected intravenous drug users (IDUs) and MSMs in Guangzhou, China. We further characterized a novel HIV-1 URF isolated from an HIV-infected MSM. Near full-length genome (NFLG) phylogenic analysis showed that this novel URF was composed of CRF07_BC and CRF55_01B. Our work demonstrates that monitoring the genetic evolution of HIV-1 will provide vital insights into our understanding of the dynamics and complexity of the HIV-1 epidemic in China. This, in turn, will provide critical information about HIV-1 replication, rational design of optimal therapeutic regimens for HIV-1-infected patients, and future vaccine development in China.

## Materials and methods

### Ethics statement

Written informed consent was obtained from individuals enrolled in this study. The Ethics Committee of Guangdong Provincial Dermatology Hospital and Southern Medical University approved the study.

### Samples

The serum or plasma samples were collected in cross-sectional studies during January to June, 2013 from HIV-1-infected individuals including 59 IDUs and 124 MSMs in Guangzhou, China, and stored at -80°C.

### Viral RNA extraction, gene amplification and sequencing

Viral RNAs were extracted from 140μl of plasma with QIAGEN viral RNA kit (Cat:52906) according to the manufacturer’s recommendations. Subsequently, RT-nested-PCR was performed to amplify HIV-1 p17 (670 bp), pol (840 bp), and gp41 (461 bp) genes, which are at nt761-1437, nt2390-3229, and nt7840-8300, respectively based on HIV-1 HXB2 numbering [17]. After purification, PCR fragments were sequenced by ABI PRISM 3730XL DNA Analyzer (Applied Biosystems, USA). The PCR primers and conditions for HIV-1 RNA detection and genotyping have been reported previously [18]. The information of the primer sets for the NFLG sequence of HIV-1 were described in the supporting material (S1 Table). The NFLG sequence reported in this study has been deposited in the GenBank database (accession number: KY201177).

### Sequence analysis

A phylogenetic analysis is performed to determine HIV-1 subtype using MEGA version 6.0 [19]. Nucleotide sequences are aligned by the Clustal W program. Neighbour-joining trees are reconstructed with 1000 bootstrap replicates. Pair wise evolutionary distances is calculated with Kimura’s two parameters; HIV reference subtypes and CRFs are downloaded from Los Alamos HIV Sequence Database (https://www.hiv.lanl.gov/)). Discordant gene regions or outlier positions in the trees are further analyzed using the jumping profile Hidden Markov Model program (jpHMM; http://jphmm.gobics.de/)). The similarity between HIV sequences is plotted using SimPlot 3.5.1 software [20]. The schematic structure is created using the Recombinant HIV-1 Drawing Tool (www.hiv.lanl.gov/content/sequence/DRAW_CRF/recom_mapper.html). Break point positions relative to HXB2 numbering are located by the HIV Sequence Locator (www.hiv.lanl.gov/content/sequence/LOCATE/locate.html).

## Results

### The genetic diversity of HIV-1 in IDUs and MSMs

The genetic diversity of HIV-1 strains was analyzed in 183 plasma samples from HIV-1 infected IDUs and MSMs. PCR fragments of HIV-1 p17 (*gag*), *pol* and gp41 (*env*) genes were successfully obtained and sequenced from 180 (98.4%), 142 (77.6%), and 182 (99.5%) samples, respectively. The genotyping results based on the sequencing data of 2 or 3 genes showed that CRF01_AE, CRF07_BC and CRF08_BC were the dominant strains and accounted for 32.2%, 27.9% and 17.5%, respectively (Table 1). HIV-1 subtype B and CRF55_01B accounted for 8.2% and 6.6%, respectively whereas only 1.1% of the samples tested was infected with HIV-1 subtype C. In addition, about 6.0% of the samples were assigned as URFs due to the discordant results of the 2 or 3 individual gene sequencing data. These URFs were the recombinants between CRF01_AE, CRF55_01B, CRF07_BC and subtype B. However, the distribution of HIV-1 genotypes was quite different in IDU and MSM population. As we can see in Table 1, the dominant genotypes in IDUs were CRF08_BC (54.2%) and CRF07_BC (22.0%) followed by subtype B (8.5%), CRF01-AE (5.1%) and subtype C (3.4%) whereas 6.8% of the IDU samples were HIV-1 URFs. HIV-1 genotypes in MSMs were dominated by CRF01_AE (45.1%) and CRF07_BC (30.6%) followed by CRF55_01B (9.6%), subtype B (8.1%), URF (5.6%) and CRF59_01B (0.8%). There was no subtype C and CRF08_BC identified in the MSMs in our study.

**Table 1:** HIV-1 Genotypes in IDU and MSM Subjects.

**HIV-1 Genotype**	**No. (%)**		
	Total	IDU^1^	MSM^2^
**CRF01_AE**	59 (32.2)	3 (5.1)	56 (45.1)
**CRF07_BC**	51 (27.9)	13 (22.0)	38 (30.6)
**CRF08_BC**	32 (17.5)	32 (54.2)	0 (0.0)
**Subtype B**	15 (8.2)	5 (8.5)	10 (8.1)
**CRF55_01B**	12 (6.6)	0 (0.0)	12 (9.6)
**CRF59_01B**	1 (0.5)	0 (0.0)	1 (0.8)
**Subtype C**	2 (1.1)	2 (3.4)	0 (0.0)
**URF^3^**	11 (6.0)	4 (6.8)	7 (5.6)
**Total**	183 (100.0)	59 (100.0)	124 (100.0)

^1^IDU, intravenous drug user

^2^MSM, mem who have sex with mem

^3^URF, unique recombinant form

### Identification and phylogenetic analysis of a novel HIV URF

One sample, from the subject GD698, was found to contain HIV-1 recombination events in HIV-1 *gag* ^*CRF07_BC*^*/pol*^*CRF07_BC*^*/env*^*CRF55_01B*^, indicating a possible novel recombinant form of CRF07_BC/CRF55_01B. The subject GD698 was a 31-year-old male, who had been recently infected with HIV-1 through homosexual contact in Guangzhou in the southern part of China. He was unmarried and treatment-naive for antiretroviral therapy. His CD4+ T cell counts and viral load were not available.

**Fig 1:**
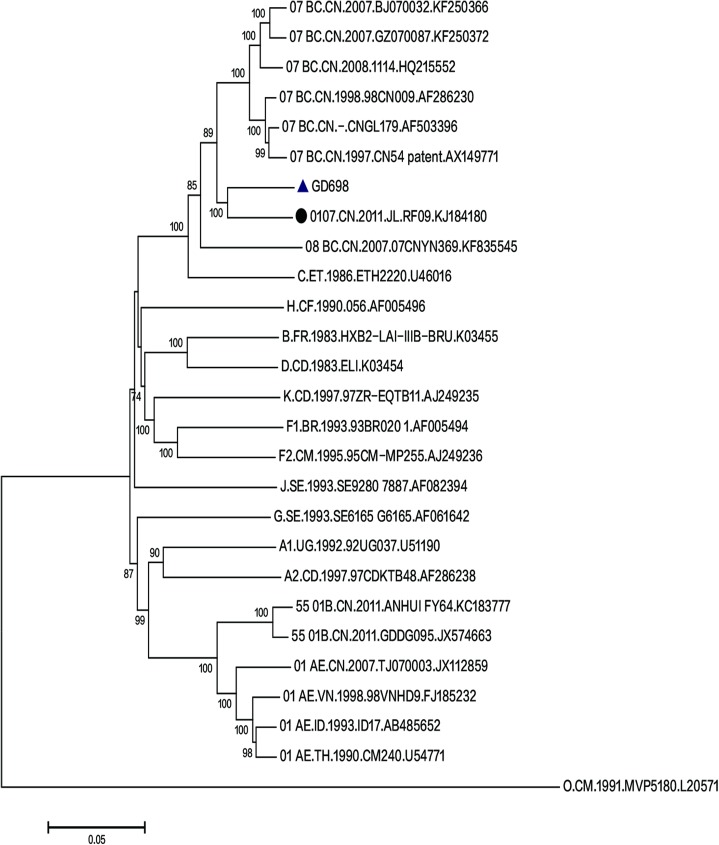
Phylogenetic analysis of the NFLG of the HIV-1 GD698 isolate. The neighbor-joining phylogenetic tree was constructed by using the MEGA 6 software package. All the reference strains of subtype A–D, F–H, J, K, CRF01_AE, CRF07_BC, CRF55_01B and CRF08_BC were retrieved from the Los Alamos National Laboratory HIV Sequence Database (http://hiv-web.lanl.gov/). The GD698 and JL.RF09 isolates are labeled with a blue solid triangle and black solid circle, respectively. The Bootstrap analysis was performed with 1,000 replications, and the bootstrap probability (more than 70%) is shown on the nodes. The scale bar represents 5% genetic distance (0.05 substitution per site).

The NFLG sequence of HIV-1 from GD698 was obtained and was 8,671 bp in length extending from nucleotides (nt) 790 to 9461 according to the HXB2 calibrator [17] and spanning the *gag*, *pol*, *env*, *tat*, *rev*, *vif*, *vpr*, *vpu*, and *nef* genes as well as the 5’ portion of the 3’ long terminal repeat (LTR). A BLAST search was performed and the possibility of cross-contamination was excluded. Moreover, the BLAST search found a high identity score of 93% between the GD698 isolate and an HIV-1 isolate JL.RF09, which is a novel recombinant form of CRF07_BC/ CRF01_AE identified in MSM in the northern part of China [21]. GD698 also showed a high identity score of 91% with several CRF07_BC isolates found in Sichuan and Xinjiang, China. Further phylogenetic analysis using the neighbor-joining method implemented in the MEGA 6 software package showed that GD698 clustered with the JL.RF09 strain to form a distinct monophyletic branch, which is distantly related to the CRF07_BC reference sequences (Fig 1). These results indicate that the parental origin of GD698 includes the CRF07_BC and CRF01_AE or CRF55_01B isolates.

### Characterization of the novel HIV-1 URF

**Fig 2:**
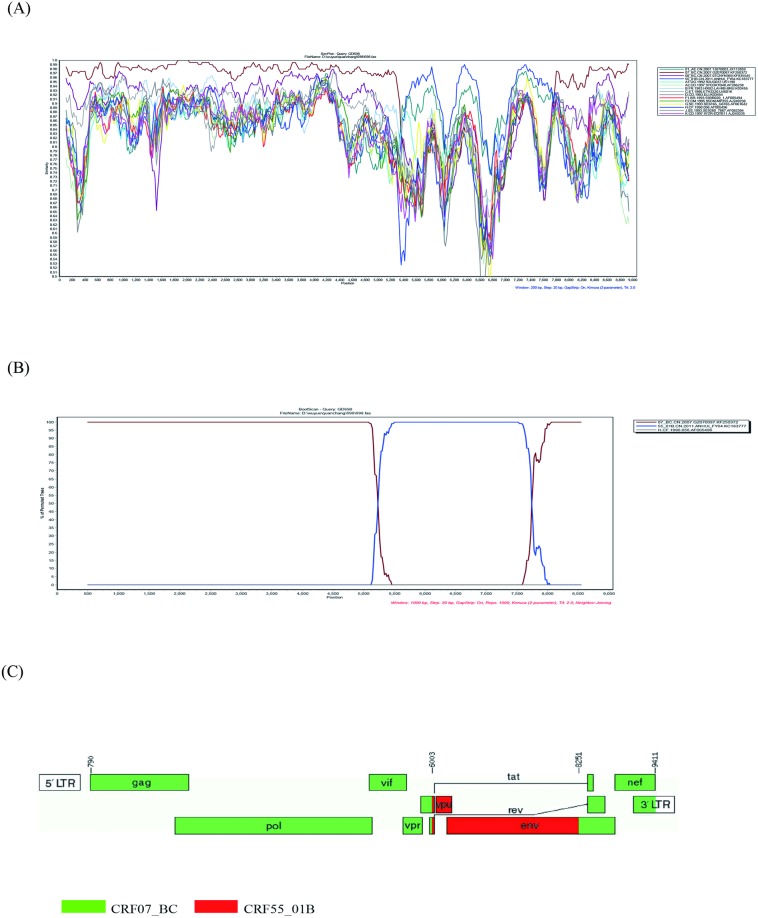
Characterization of GD698 isolate. (A) The similarity between GD698 and the reference sequences was plotted using SimPlot 3.5.1 software. (B) Bootscanning analyses of the NFLG of GD698. CRF55_01B (KC183777) and CRF07_BC (KF250372) were used as putative parental reference sequences, and subtype H (AF005496) was used as an outgroup. (C) Recombinant map results for GD698. The schematic structure was created using the Recombinant HIV-1 Drawing Tool (www.hiv.lanl.gov/content/sequence/DRAW_CRF/recom_mapper.html). Break point positions relative to HXB2 numbering were located by the HIV Sequence Locator (www.hiv.lanl.gov/content/sequence/LOCATE/locate.html).

To analyze the similarity between GD698 and HIV-1 reference strains, the sequence was submitted to SimPlot analysis for comparison with the major HIV-1 subtypes as well as CRF01_AE, CRF07_BC and CRF55_01B. The result revealed a recombinant form consisting of CRF07_BC and CRF55_01B, with two obvious break points, approximately between nt positions 5,300 and 7,800 (Fig 2A). Bootscanning analysis was subsequently carried out to position the exact recombination breakpoints by using CRF55_01B (Genbank accession number KC183777) and CRF07_BC (KF250372) as putative parental reference sequences, and subtype H (AF005496) as an outgroup (Fig 2B). Bootscanning analysis revealed two unique recombination breakpoints at nt positions 6,003 and 8,251 (relative to the HXB2 genome), which are located in the *vpu/env* and *env* genes, respectively (Fig 2B). The two breakpoints divided the NFLG of GD698 into three regions: region I (HXB2 nt 790–6,002), CRF07_BC in the HIV-1 *gag*, *pol*, *vpr* and partial *vpu* gene; region II (HXB2 nt 6,003–8,250), CRF55_01B in the HIV-1 *vpu* and the majority of *env* gene; and region III (HXB2 nt 8,251–9,411), CRF07_BC in the 3’ part of *env*, and *tat*, *ref*, *nef* genes as well as 3’LTR (Fig 2B). Similar results were obtained using online software jpHMM-HIV. The mosaic map of GD698 was then generated using the Recombinant HIV-1 Drawing Tool (www.hiv.lanl.gov/content/sequence/DRAW_CRF/recom_mapper.html) and is shown in Fig 2C. The recombinant structure is clearly distinct from any known CRFs or URFs reported to date.

**Fig 3:**
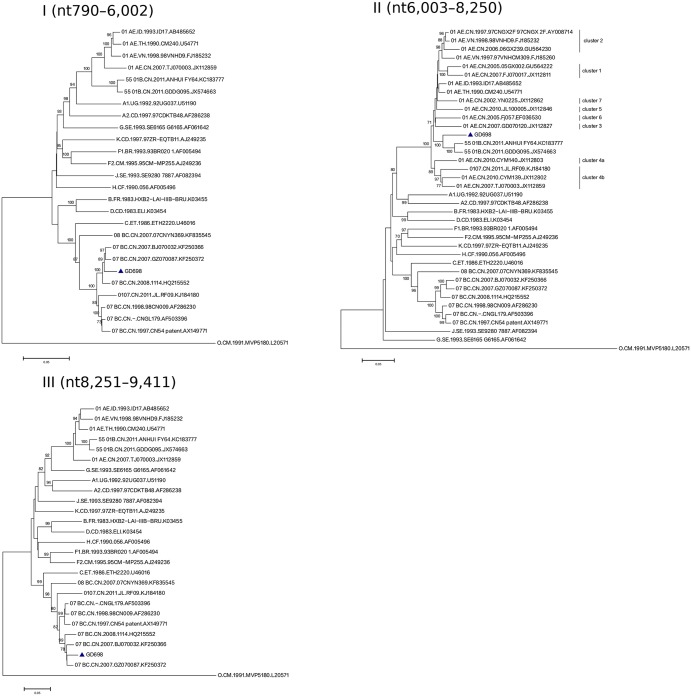
Phylogenetic analysis of GD698 regional segments. Regions of the sequence alignment were extracted according to the indicated breakpoints. Each segment was analyzed separately with the phylogenetic neighbor-joining method with the bootstrap value of > 70%. Representative trees are illustrated for regions I through III. Loci of genomic segments are based on the HXB2 numbering engine. The genetic distance corresponding to the lengths of the branches is shown by the bottom line.

Furthermore, the subregion tree analysis of GD698 indicated that at region I, GD698 was clustered together with the CRF07_BC isolates identified in MSMs, whereas the HIV-1 isolate JL.RF09 clustered with CRF07_BC strains isolated from IDU in China (Fig 3). At region II, GD698 fell into the CRF55_01B cluster whereas HIV-1 isolate JL.RF09 was within the cluster 4b of CRF01_AE, although both GD698 and JL.RF09 were within the CRF01_AE clade (Fig 3). The different location may represent the different parental origin of these two isolates. It has been reported that the CRF01_AE regions within CRF55_01B are from Thailand CRF01_AE and are not related to the CRF01_AE variants identified among MSMs in China [22]. At region III, the classification of GD698 was similar to that at region I, but was only distantly related to the HIV-1 isolate JL.RF09. The subregion tree analysis further confirmed the two recombinant breakpoints identified by bootscanning analysis and the subtle difference between GD698 and HIV-1 isolate JL.RF09. It also demonstrated that the CRF07_BC fragments (region I and III) in GD698 belong to the MSM-related subcluster. In addition, region II of GD698 was more like the sequence of CRF55_01B (bootstrap value 98%), rather than CRF01_AE, which commonly exists in MSMs.

**Fig 4:**
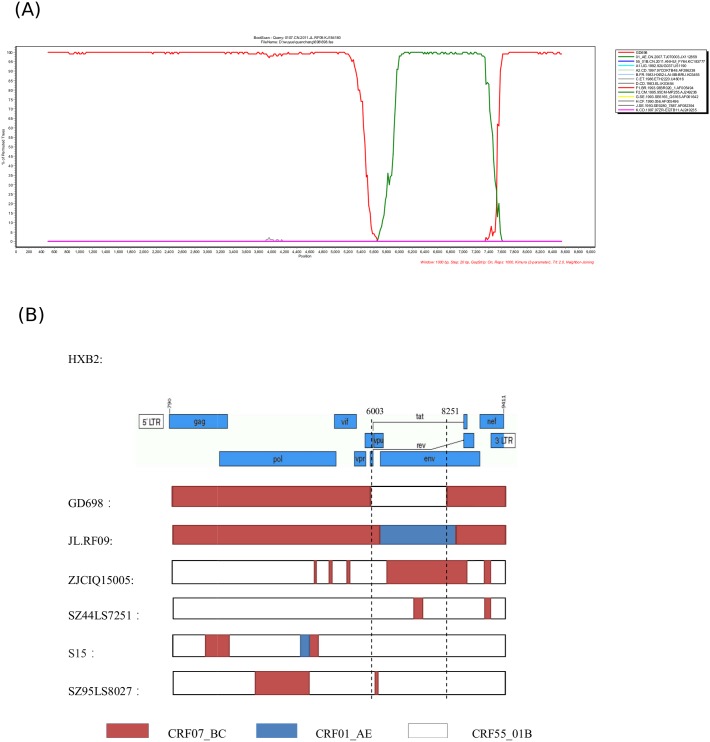
Comparison between GD698 and other recombinants of CRF07_BC/ CRF55_01B. (A) Bootscan analysis of JL.RF09 queried against GD698. Bootscan analysis was performed using SimPlot 3.5.1 software configured with 1000 bootstrap replicates, a 1000 bp window, and a step size of 50 bp. The x-axis shows all aligned nt of the sequence analyzed. The y-axis shows the bootstrap value. (B) Diagram of the genomic structure of CRF07_BC/CRF55_01B containing recombinants. HBX2 genomic regions are indicated at the top of the plot as the reference structure. Breakpoint locations are based on the HXB2 numbering. SimPlot and Genotyping were used to analyze each of the resulting genomes separately; recombinant structures of each strain were determined and listed. The red bar indicates the CRF07_BC regions, the blue bar indicates the CRF01_AE, and the white bar indicates CRF55_01B.

The difference between the HIV-1 isolates GD698 and JL.RF09 was further analyzed by bootscanning using the SimPlot software package. When queried against the GD698 sequence, JL.RF09 did not cluster phylogenetically with GD698 in the *env* region, but was more closely related to CRF01_AE and not CRF55_01B (Fig 4A). These results are consistent with the previous definition of the JL.RF09 isolate as a recombinant of CRF07_BC/CRF01_AE [21]. Furthermore, the difference between GD698 and other CRF07_BC/CRF55_01B containing recombinants was observed based on the different mosaic structures of their genomes (Fig 4B)

In summary, the genetic diversity of HIV-1 in both IDUs and MSMs in Guangzhou, China was determined and found to be dominated by CRF01_AE, CRF07_BC and CRF08_BC. We then characterized a NFLG sequence from an individual in the population of MSM in Guangzhou, China, and identified this sequence as a novel third-generation HIV-1 recombinant of CRF07_BC and CRF55_01B.

## Discussion

CRF07_BC was first described in 2000 [3], but may have come from western Yunnan, China in the early 1990s [23]. CRF55_01B was first identified in 2012 [5], but its origin and spread could be traced back to HIV-1-infected MSM in 2001 [24]. Zhao et al reported that from 2006 to 2012, the prevalence of HIV-1 CRF07_BC and CRF55_01B rapidly increased from 12.5% and 0% in 2006 to 43.2% and 16.0% in 2012, respectively [25]. Our results and the previous studies indicate that CRF07_BC and CRF55_01B are now the dominant genotypes of HIV-1 in the MSM population. Novel recombinants between the two HIV-1 dominant genotypes of CRF07_BC and CRF55_01B are emerging [14–16]. For example, Wang et al. reported a novel HIV-1 recombinant composed of CRF55_01B as the backbone and CRF07_BC with 12 recombinant break points observed in the *pol*, *vif*, *vpr*, *tat*, *rev*, *env*, *nef*, and 3’ LTR regions from a Malaysian immigrant worker ZJCIQ15005 in Zhejiang, China [14]. Jia et al. reported a novel recombinant in which two small fragments of CRF07_BC are inserted into the backbone sequence of CRF55_01B from heterosexual persons SZ44LS7251 and SZ95LS8027 in Shenzhen, China [15]. In addition, Gui et al. reported a novel recombinant strain S15 with backbone sequence of CRF55_01B and the fragments of CRF07_BC in both the *gag* and *pol* genes from a MSM in Shenzhen, China [16]. However, the mosaic structures of these URFs are quite different, and did not share a common ancestor (Fig 4B). Importantly, these results indicate that active recombination is occurring between the two dominant HIV-1 genotypes CRF07_BC and CRF55_01B in the MSM population in China.

However, little is known about the characteristics of the CRF07_BC, CRF55_01B virus, and the emerging recombinant forms of CRF07_BC/CRF55_01B, and their effect on disease progression among the HIV-1-infected individuals. Yu et al. reported that CRF07_BC isolates were about 5-fold less sensitive than HIV-1 subtype B’ isolates to entry inhibitors enfuvirtide, maraviroc and TAK779, and showed no susceptibility to HIV entry inhibitors AMD3100 [26]. It has been reported that around 25%-30% of the CRF07_BC viruses found in the MSM and IDU population contain a unique, signature 7 amino-acid deletions between the highly conserved PTAP and LXXLF motif, which are the Tsg101and Alix binding domains in the p6 protein of HIV-1 *gag*, respectively [27–29]. The specific deletion mutation may affect the interaction between the HIV-1 p6 protein and Alix, and virus budding. Furthermore, the 7 amino-acid deletions in the HIV-1 *pol* gene may further impair the activity of the HIV-1 protease protein. It has been found that the IDUs infected with the CRF07_BC variant had significantly lower viral loads and slower immunological progression [30, 31]. The specific CRF07_BC variant showed lower replication capacity and decreased virus growth kinetics likely due to poorer protease-mediated processing and slower viral maturation processes [29–31], but it should be noted that the results regarding the effect of the 7-amino acid deletion on HIV-1 viral load and CD4 counts are controversial [27, 28]. These results suggest that the novel CRF07_BC virus and its variant may have different replication capacities which in turn would impact the disease progression and therapy.

In conclusion, our work demonstrates that monitoring the genetic evolution of HIV-1 and investigating the novel properties of these newly emerging inter-CRF recombinants will provide vital insights into our understanding of the dynamics and complexity of the HIV-1 epidemic in China. This, in turn, will provide critical information about HIV-1 replication, rational design of optimal therapeutic regimens for HIV-1-infected patients, and future vaccine development in China.

## Sequence data

The NFLG sequence of GD698 has been deposited in GenBank with the accession number KY201177.

## Supporting information

S1 Table List of PCR primers used for amplification of the near full-length genomes of HIV GD698 (DOCX)
