# Supplementary material for: Characterization of a novel HIV-1 unique recombinant form between CRF07_BC and CRF55_01B in men who have sex with men in Guangzhou, China
Source: PLoS One. 2017 Apr 12;12(4):e0175770. doi: 10.1371/journal.pone.0175770 (PMC5389846; doi:10.1371/journal.pone.0175770)
Supplement: S1 Table — (DOCX) [file pone.0175770.s001.docx]

**S1 Table. List of PCR primers used for amplification of the near full-length genomes of HIV GD698**

|  | Primer | Reaction | Sequence | HXB2 coordinates | Size (bp) |
| --- | --- | --- | --- | --- | --- |
| 1 | gag outer F | 1st PCR | GCTGAAGCGCGCACGGCAAGAG | 760-2530 | 1771 |
|  | gag outer R |  | ACCTTTGGTCCATCCATTCCTG |  |  |
|  | gag inner F | 2nd PCR | TTTGACTAGCGGAGGCTAGA |  |  |
|  | gag inner R |  | CAATCTGAGTCAACATATTTCGTCC |  |  |
| 2 | ANA corr | 1st PCR | CAGGAGCAGATGATACAGTATTAG | 2390-3229 | 840 |
|  | SP5R |  | ATTTATCAGGATGGAGTTCA |  |  |
|  | AOA Corr | 2nd PCR | GATAGGGGGAATTGGAGG |  |  |
|  | SP5F-r |  | AATGGAGGTTCTTTCTGATGT |  |  |
| 3 | 651 outer F | 1st PCR | ATAGGGCAGCATAGAGCAAAAATAG | 3204-6353 | 3150 |
|  | 651 outer R |  | TTCCAGGTGTATTTCTTGTGGGTTG |  |  |
|  | 651 inner F | 2nd PCR | AGAAACATCAGAAAGAACCTCCATT |  |  |
|  | 651 inner R |  | CAGGAACCCCATAATAAACTGTAAC |  |  |
| 4 | env-1 outer F | 1st PCR | AGTGCAATAGTAGGACTGATAGTAGCG | 6208-8309 | 2101 |
|  | gp41R1 |  | AACGACAAAGGTGAGTATCCCTGCCTAA |  |  |
|  | env-1 inner F | 2nd PCR | GCAGAAGACAGTGGAAATGAGAGTG |  |  |
|  | gp47R1 |  | TTAAACCTATCAAGCCTCCTACTATCATTA |  |  |
| 5 | gp40F1 | 1st PCR | TCTTAGGAGCAGCAGGAAGCACTATGGG | 7849-9546 | 1698 |
|  | env-2outer R |  | CTTTATTGAGGCTTAAGCAGTGGG |  |  |
|  | gp46F2 | 2nd PCR | ACAATTATTGTCTGGTATAGTGCAACAGCA |  |  |
|  | env-2inner R |  | GAGAGACCCAGTACAGGCAAAAAGC |  |  |
